# Epidemiology of community origin of major multidrug-resistant ESKAPE uropathogens in a paediatric population in South-East Gabon

**DOI:** 10.1186/s13756-023-01250-y

**Published:** 2023-05-12

**Authors:** Yann Mouanga-Ndzime, Richard Onanga, Neil-Michel Longo-Pendy, Michelle Bignoumba, Cyrille Bisseye

**Affiliations:** 1 Unité de Recherche et d’Analyses Médicales, Laboratoire de Bactériologie, Centre Interdisciplinaire de Recherches Médicales de Franceville, BP 769, Franceville, Gabon; 2 Laboratoire de Biologie Moléculaire et Cellulaire (LABMC), Université des Sciences et Techniques de Masuku, BP 943, Franceville, Gabon; 3 Unité de Recherche en Ecologie de La Santé (URES), Centre Interdisciplinaire de Recherches Médicales de Franceville, BP 769, Franceville, Gabon

**Keywords:** Paediatric UTIs, *ESKAPE*, Antibiotic resistance, South-East Gabon

## Abstract

**Background:**

Urinary tract infections (UTIs) in children are very common. They are often associated with a high risk of sepsis and death. In recent years, antibiotic-resistant uropathogens *ESKAPE* (*Enterococcus faecium*, *Staphylococcus aureus*, *Klebsiella pneumoniae*, *Acinetobacter baumannii*, *Pseudomonas aeruginosa*, and *Enterobacteriaceae*) are increasingly encountered in UTIs. These bacteria, usually multidrug-resistance (MDR), extensive drug-resistance (XDR), pandrug-resistance (PDR), Extended-spectrum cephalosporin-resistance (ESC), Usual Drug Resistance (UDR), Difficult-to-Treat Resistance (DTR) and Carbapenem-resistance Enterobacteriales (CRE), represent a global threat for the management of paediatric UTIs. The aim of this study was to determine the epidemiology of community origin and antibiotic sensitivity of major *ESKAPE* uropathogens in paediatric UTIs in South-East Gabon.

**Methods:**

The study involved 508 children aged 0–17 years. Identification of bacterial isolates was carried out using Vitek-2 compact automated system and the antibiogram with the disk diffusion and microdilution methods according to the European Committee on Antimicrobial Susceptibility Testing recommendations. Logistic regression analysis was used to assess the impact of patients' socio-clinical characteristics on uropathogens phenotype in both univariate and multivariate analysis.

**Results:**

The prevalence of UTIs was 59%. *E. coli* (35%) and *K. pneumoniae* (34%) were the main *ESKAPE* involved in UTIs followed by *Enterococcus* spp. (8%) and *S.* aureus (6%). Among major *ESKAPE*, DTR-*E. coli* (*p* = 0.01), CRE-*E. coli* (*p* = 0.02) and XDR-*E. coli* (*p* = 0.03), Trimethoprim-sulfamethoxazole-resistant bacteria (*p* = 0.03) were associated with abdomino-pelvic pain. While MDR-*E. coli* (*p* < 0.001), UDR-*E. coli* (*p* = 0.02)*,* ESC-*E. coli* (*p* < 0.001), MDR- *Enterococcus* (*p* = 0.04)*,* UDR- *Enterococcus* (*p* = 0.02), bacteria resistant to Ampicillin (*p* < 0.01), Cefotaxime (*p* = 0.04), Ciprofloxacin (*p* < 0.001), Benzylpenicillin (*p* = 0.03) and Amikacin (*p* = 0.04) were more frequent among male children. MDR-*Enterococcus* (*p* < 0.01), bacteria resistant to Amoxicillin-clavulanic acid (*p* = 0.03), Cefalotin (*p* = 0.01), Ampicillin (*p* = 0.02) and Gentamicin (*p* = 0.03) were associated with treatment failure. In addition, Trimethoprim-sulfamethoxazole-resistant bacteria (*p* = 0.03) was associated with recurrent UTIs while those resistant to Ciprofloxacin was associated with pollakiuria (*p* = 0.01) and urinary burning (*p* = 0.04). Furthermore, UDR-*K. pneumoniae* (*p* = 0.02) was more frequent in neonates and infants.

**Conclusion:**

This study determined the epidemiology of *ESKAPE* uropathogens in paediatric UTIs. It found a high prevalence of paediatric UTIs associated with children’s socio-clinical characteristics and diverse bacterial antibiotic resistance phenotypes.

## Introduction

In human medicine, urinary tract infections (UTIs) are the second most common infection worldwide. It contributes significantly to morbidity and mortality in outpatient and inpatient settings representing approximately 20 to 60% of all infections [1, 2].

In a paediatric population, an estimated 7% of girls and 2% of boys will develop at least one UTI by age 6 [3]. Paediatric UTIs are common and often associated with a high risk of sepsis and death [4]. In most cases, the symptoms are nonspecific, especially in newborns and infants [5]. Recurrences are frequent (30% to 40% of cases), renal scarring is not uncommon and can lead to long-term high blood pressure and nephron reduction [6]. Infection is fostered by the presence of a functional or organic abnormality responsible for colonisation of the bladder, urinary stasis or reflux into the upper urinary tract [5]. Normal GI flora is usually the reservoir of bacteria found in urinary tract infections [7].

Overall, the common causative agents of UTIs are Gram-negative bacteria, mainly *Escherichia coli* (*E. coli*) followed by *Klebsiella pneumoniae* (*K. pneumoniae*) and *Proteus mirabilis*; while *Enterococcus faecalis* is the most common Gram-positive bacteria [8]. However, the epidemiology and distribution of uropathogenic species have shown strong geographical and temporal variations but also a relationship with the patient populations studied [9]. An earlier study showed that the aetiology of UTIs has evolved significantly in hospital and community settings [10]. There is an increasing switch to “less common” micro-organisms with more pronounced roles including pathogens such as *Enterococcus faecalis* (E), *Staphylococcus aureus* (S), AMR-*Klebsiella pneumoniae* (K), *Acinetobacter baumannii* (A), *Pseudomonas aeruginosa* (P) and *Enterobacteriaceae* (E) known as *ESKAPE* group [10]. Through its overall induced mortality and economic impact, the *ESKAPE* group represents the greatest clinical challenge for antibiotic resistance surveillance and management interventions [11]. These bacteria are considered a priority by the World Health Organization (WHO) in the global monitoring of antibiotic resistance [12]. Indeed, *ESKAPE* pathogens frequently exhibit acquired resistance to a variety of antimicrobial agents, such as oxazolidinones, lipopeptides, macrolides, fluoroquinolones, tetracyclines, β-lactams (including carbapenems and combinations of beta-lactamase inhibitors) [13]. These acquired resistances result in the emergence of *ESKAPE* multidrug-resistance (MDR), extensive drug-resistance (XDR), pandrug-resistance (PDR), Extended-spectrum cephalosporin-resistance (ESC) and Carbapenem-resistant Enterobacteriales (CRE). In paediatrics, the situation is rather worrying because many antibiotic molecules available for adults (quinolones, fosfomycin, nitrofurantoin, mecilinam, etc.) are contraindicated in children or do not have a marketing authorisation or paediatric dosage form [5]. In addition, antibiotic resistance of UTI pathogens isolated from children is increasing, especially for commonly used antibiotics [14]. In South-East Gabon, a previous study on UTIs showed high frequency of resistance of bacteria to antibiotics in children under 5 years [15]. The choice to study *ESKAPE* pathogens isolated from paediatric UTIs is justified by the state of the Gabonese health system, which is confronted with both a glaring shortage of paediatricians and clinical microbiology laboratories.

The aim of this study was to determine the epidemiology and antibiotic sensitivity of major *ESKAPE* uropathogens in community-acquired paediatric urinary tract infections in Southeastern Gabon.

## Methods

### Design, study area and population

The study was conducted from January 2018 to December 2021. It involved all children aged 0 to 17 years, identified in the community as requesting a cyto-bacteriological examination of urine (CBEU) by the only microbiology laboratory in the city of Franceville, capital of the Haut-Ogooué province in the South-East of Gabon, bordering the Republic of Congo. During the study period a single paediatrician was practicing in the city.

Children included in the study were stratified into four paediatric populations: neonates and infants (0–2 years), early childhood (3–6 years), late childhood (7–12 years) and adolescence (13–17 years).

### Inclusion criteria

All non-hospitalised children of both sexes referred to the Microbiology Laboratory for a CBEU were eligible for inclusion in this study.

### Sample collection and data collection

For individual children, urine samples were collected as previously described [15]. For children who were not able to use the toilet alone, urine collection by 'clean capture' was preferred. Otherwise, urine was collected in a sterile adhesive collection bag with the help of parents, carers or a nurse.

The urine bottle was sealed, identified, indicating the time of collection and then transported to the laboratory at room temperature. The sociodemographic and clinical data of each patient was collected through a structured questionnaire.

### Culture and identification of bacterial isolates

The bacterial culture consisted of aseptically inoculating ten microliters (10 μL) of total urine using a sterile single-use loop in the level 2 microbiological safety station. The inoculation was carried out systematically on Agar Media, CLED (Cystine-Lactose-Electrolyte-Déficient, bioMérieux, Marcy-l’Étoile, France), Mac Conkey (McC, bioMérieux, Marcy-l’Étoile, France) and COS (Columbia agar + 5% sheep blood, bioMérieux, Marcy-l’Étoile, France). Urine samples were inoculated within two (2) hours of collection to avoid contamination. The inoculated media were incubated aerobically in a bacteriological incubator at 35 °C for 18 to 24 h. According to Kass criteria, a number ≥ 10^5^ colony forming units (CFU)/mL was considered positive; a colony number < 10^5^ CFU/mL or with more than two (2) types of bacterial colonies were considered contamination [16]. The bacterial count was done independently of the sex of the patient and of the pathogen isolated.

Presumptive identification of *ESKAPE* isolates was made after Gram stain, oxidase test to differentiate fermentative from non-fermentative Gram-negative bacilli, catalase test to discriminate genus *Staphylococcus* from genera *Streptococcus* and *Enterococcus.* Conventional biochemical tests (automated VITEK-2 system, bioMérieux, Marcy-l'Étoile, France) allowed the complete identification of bacterial genera, species and subspecies. The procedure for sample preparation and identification by VITEK-2 has been described in a previous study [17].

### Antibiotic sensitivity test

The antibiotic sensitivity of *ESKAPE* isolates was determined by the diffusion disc method (Kirby-Bauer) on Mueller–Hinton (MH) agar (bioMérieux, Marcy-l'Étoile, France) according to the recommendations of the European Committee on Antimicrobial Susceptibility Testing (EUCAST) [18]. Briefly, MH agars were seeded with a standardised suspension (0.5 McFarland) of each *ESKAPE* isolate from the 24-h primary cultures. Antibiotic discs (Oxoid, Basingstoke Hampshire, UK) were firmly placed on the surface of the seeded plates. The culture media were then incubated at 35 °C for 24 h. The inhibition diameters around each antibiotic were interpreted according to EUCAST.

For antibiotics requiring microdilution methods, the minimum inhibitory concentrations (MIC) were measured on VITEK 2 Compact-AST (bioMérieux, Marcy-l'Étoile, France).

For Gram-negative isolates, the following antibiotic discs were used: Ampicillin, Amoxicillin-clavulanic acid, Piperacillin-tazobactam, Ticarcillin, Cefalotin, Cefoxitin, Cefotaxime, Ceftazidime, Cefepime, Ertapenem, Imipenem, Gentamicin, Tobramycin, Amikacin, Nalidixic acid, Ofloxacin, Ciprofloxacin, Nitrofurantoin and Trimethoprim-sulfamethoxazole.

The panel of antibiotics used for Gram-positive isolates was: Moxifloxacin, Erythromycin, Clindamycin, Quinupristin-dalfopristin, Linezolid, Vancomycin, Tetracycline, Tigecycline, applicable to all Gram-positive bacteria.

For *Enterococcus* spp., Ampicillin and Nitrofurantoin were also tested while the antibiotics Benzylpenicillin, Oxacillin, Gentamicin, Ciprofloxacin, Levofloxacin and Trimethoprim-sulfamethoxazole were additionally tested on *Staphylococcus aureus* isolates.

### Classification of antibiotic resistance phenotypes

Resistance phenotypes have been classified into seven categories: Multi-drug Resistant (MDR), Extensive Drug Resistant (XDR), Pandrug-resistant (PDR), Usual Drug Resistance (UDR), Difficult-to-Treat Resistance (DTR), Extended-spectrum cephalosporin-resistant (ESC), Carbapenem-resistant Enterobacterales (CRE). These different categories of resistance have been defined in several previous studies [10, 19, 20].

Multiple antibiotic resistance (MAR) index was determined for each isolate using the formula MAR = n/N, where n represents the number of antibiotics to which the test isolate showed resistance and N represents the total number of antibiotics to which the test isolate has been evaluated [21]. The MAR index ranged from 0.0 to 1.0.

### Statistical analysis

The chi-square test was used to compare the prevalence of different phenotypes of each bacterium in the study population. In addition, the co-occurrence of resistance between different families of antibiotics (Beta-lactams, Quinolones, Aminoglycosides, Sulfonamides and Nitrofurans) was highlighted by a Venn diagram with the "Venn Diagram" package version 1.7.3. To visualize the data, a Factorial correspondence analysis (FCA) was performed to reduce the dimensions of the dataset with the packages FactoMineR and Factoextra. In this process, the data were projected onto a two-dimensional space, namely Dimension 1 [Dim 1] and Dimension 2 [Dim 2], where the original data had the greatest explained variance. Typically, Dim 1containing the largest variance in the data projections, followed by Dim 2.

Logistic regression was used to assess the impact of patients' socio-clinical characteristics on resistant uropathogens phenotypes in both univariate and multivariable analysis. Odds ratios (OR) and 95% confidence intervals are presented. All statistical analyses and graphs were performed with R software, version 4.0.2 and SPSS version 20 (IBM, USA). *P* values < 0.05 were considered statistically significant.

## Results

### Socio-clinical characteristics of study patients

A total of 608 urine samples was collected over a 4-year period, of which 100 were excluded from the study because their socio-clinical parameters were not fully documented. Males represented 56% of the study population (289/508). The male/female sex ratio was 1.31. The patients’ mean age was 3.96 ± 4.67 years, and 57% (293/508) were neonates and infants. A large majority of patients were from urban areas (78%, 397/508), with an urban/rural ratio of 3.57.

Regarding the clinical signs observed in the latter, 43% (220/508) had fever while 42% (215/508) had a history of pre-emptive antibiotic therapy (Table 1).

**Table 1:** Patients' socio-clinical parameters, stratified by confirmed UTI cases within each grouping

Characteristics	Total number (n = 508)	UTIs (n = 304)	Percentage (%)	*p*-value
**Pediatric population**				
Neonates and infants	293	229	78.2	< 0.0001
Early childhood	99	37	37.4	
Late childhood	59	14	23.7	
Adolescents	57	24	42.1	
**Sex**				
Male	289	168	58.1	NS
Female	219	136	62.1	
**Residence**				
Urban	397	247	62.2	0.05
Rural	111	57	51.4	
**Symptoms**				
Urinary signs	215	98	45.6	
Voiding burns	68	39	57.3	
Pollakiuria	40	21	52.5	
Abdomino-pelvic pain	57	18	31.6	NS
Hematuria	8	3	37.5	
Low back pain	33	13	39.4	
Incontinence	9	4	44.4	
Non-urinary signs	293	206	70.3	
Fever	220	134	60.9	
Others	73	72	98.6	< 0.0001
**History of antibiotic therapy**				
Probabilistic ATB	215	111	51.6	0.001
Non- Probabilistic ATB	293	193	65.9	

*P* values were calculated using a Chi-squared test in R software, version 4.0.2

*NS* not significant

### Prevalence of urinary tract infections in community paediatrics

The overall prevalence of UTIs was 59% (304/508). UTIs were associated with age in the paediatric population. They were significantly more frequent in neonates and infants compared to other age group (78.2%; *p* < 0.001) (Table 1). Male and female children were similarly affected by UTIs (Table 1).

Regarding residence, UTIs were not significantly more prevalent in patients from urban areas than those from rural areas (62.2% vs 51.4%; *p* = 0.05).

Non-febrile children were more susceptible to UTIs (98.6% vs 60.9; *p* < 0.0001) (Table 1).

### Bacteriological profile of UTIs

UTIs were significantly associated with uropathogens in the *ESKAPE* group compared to the non-*ESKAPE* group (89% vs 11%; *p* < 0.0001). Among the *ESKAPE* pathogens, the *ESKAPE*-Gram-negative group was significantly more prevalent than the *ESKAPE*-Gram-positive group (75% vs 14%; *p* < 0.001) (Table 2). Within the *ESKAPE*-Gram-negative uropathogens, the predominant bacteria were *E. coli* (46%, 105/229) and *K. pneumoniae* (45%, 104/229).

**Table 2:** Prevalence of uropathogens isolated from children UTIs

Urinary tract infections	Total number (n = 304)	Percentage (%)
Uropathogens		
*ESKAPE*-Gram-negative	229	75
*Escherichia coli*	105	35
*Klebsiella pneumoniae*	104	34
*Pseudomonas aeruginosa*	1	0.3
*Acinetobacter baumannii*	2	0.7
*Citrobacter-Enterobacter-Serratia* (CES)	11	4
*Proteus mirabilis*	6	1
*ESKAPE*-Gram-positive	43	14
*Staphylococcus aureus*	18	6
*Enterococcus* spp.	25	8
Non-*ESKAPE*	32	11
*Morganella morganii ssp morganii*	2	0.7
*Staphylococcus coagulase negative*	23	8
*Streptococcus* spp.	2	0.7
*Micrococcus lentus*	1	0.3
*Candida albicans*	4	1.3

Among the *ESKAPE*-Gram-positive uropathogens, *Enterococcus* spp., and *Staphylococcus aureus* accounted for 58% (25/43) and 42% (18/43), respectively (Table 2).

### Distribution of resistance phenotypes in major uropathogenic Gram-negative strains

Among *E. coli* and *K. pneumoniae* isolates, ESC (65%, 136/209), MDR (64%, 135/209), and UDR (52%, 108/209) resistance phenotypes were more frequent compared to other phenotypes (Table 3).

**Table 3:** Prevalence of major *ESKAPE*-Gram- uropathogens according to resistance categories

	Resistance categories									
	MDR (n = 135)	Non-MDR (n = 74)	XDR (n = 9)	Non-XDR (n = 200)	ESC (n = 136)	Non-ESC (n = 73)	CRE (n = 11)	Non-CRE (n = 198)	UDR (n = 108)	DTR (n = 5)
*E. coli*	59 (44%)	46 (62%)	6 (67%)	99 (49%)	62 (46%)	43 (59%)	3 (27%)	102 (52%)	60 (56%)	5 (100%)
*K. pneumoniae*	76 (56%)	28 (38%)	3 (33%)	101 (51%)	74 (54%)	30 (41%)	8 (73%)	96 (48%)	48 (44%)	0 (0%)
Chi-squared	3.8	7.8	0.89	0.01	1.8	3.9	2.9	0.25	2.2	6.4
df	1	1	1	1	1	1	1	1	1	1
*P*-value	0.05	0.005	NS	NS	NS	0.04	NS	NS	NS	0.01

*P*-values were calculated using a Chi-squared test in R software, version 4.0.2

*NS* not significant

The frequency of MDR-*K. pneumoniae* strains was similar to that of MDR-*E. coli* (56% vs 44%; *p* = 0.05), while DTR-*E. coli* was significantly more prevalent than DTR-*K. pneumoniae* (100% vs 0%, *p* = 0.01). No statistically significant differences were observed when comparing the other phenotypes (Table 3).

**Fig. 1:**
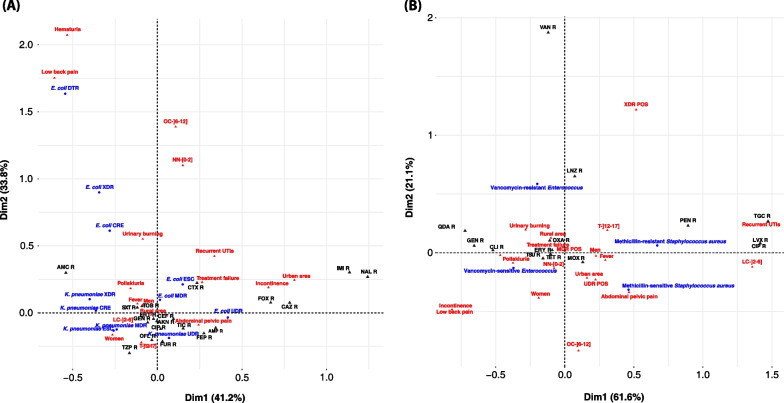
Factorial correspondence analysis (FCA) of data for major *ESKAPE* UTIs isolates according to socio-clinical parameters. Dim 1 and Dim 2 axes explained 75% of the total variance in the dataset of major *ESKAPE*-Gram-negative against 82,7% for major *ESKAPE*-Gram-positive

The association between resistant uropathogens and patients' socio-clinical characteristics was determined using factorial correspondence analysis (Fig. 1) and multivariate logistic regression (Tables 4 and 5).

**Table 4:** Multivariate analysis of bacterial resistance phenotypes according to the socio-clinical characteristics of children

Bacterial phenotypes	Socio-clinical characteristics	
	AOR (95% CI)	*P*-value
	Male	
MDR_*E. coli*	3.2 (1.7–6.0)	< 0.001
UDR_*E. coli*	2.0 (1.1–3.8)	0.02
ESC_*E. coli*	3.5 (1.8–6.7)	< 0.001
MDR_*Enterococcus*	0.3 (0.1–1.0)	0.04
UDR_*Staphylococcus*	0.2 (0.03–0.8)	0.02
	Neonates and infants	
UDR_*K. pneumoniae*	0.06 (0.006–0.6)	0.02
	Urinary burning	
UDR_*K. pneumoniae*	0.2 (0.07–0.7)	0.01
	Abdominal pelvic pain	
DTR_*E. coli*	0.0 (0.0–0.2)	0.01
CRE_*E. coli*	0.2 (0.001–0.5)	0.02
XDR*_E. coli*	0.2 (0.0–0.7)	0.03
	Treatment failure	
VSE	0.18 (0.06–0.57)	< 0.01
MDR_*Enterococcus*	0.18 (0.06–0.52)	< 0.01
UDR_*Enterococcus*	0.28 (0.09–0.83)	0.02

*AOR* adjusted Odd ratio, *CI* confident interval

**Table 5:** Multivariate analysis of bacterial antibiotic resistance according to socio-clinical characteristics of children

Antimicrobial resistance	Socioclinic characteristics	
	AOR (95% CI)	*P*-value
	Male	
Ampicillin	2.69 (1.37–5.3)	< 0.01
Ciprofloxacin	4.18 (1.94–9.01)	< 0.001
Cefotaxime	1.77 (1.008–3.12)	0.04
Benzylpenicillin	0.17 (0.03–0.85)	0.03
Amikacin	4.9 (1.04–23.3)	0.04
	Neonates and infants	
Cefalotin	0.04 (0.007–0.27)	< 0.01
Cefotaxime	0.60 (0.009–0.38)	< 0.01
Benzylpenicillin	21.29 (1.99–227.51)	0.01
	Early childhood	
Cefalotin	0.11 (0.015–0.871)	0.03
	Late childhood	
Cefalotin	0.04 (0.005–0.43)	< 0.01
Cefotaxime	0.08 (0.009–0.82)	0.03
	Urinary burning	
Ciprofloxacin	0.30 (0.09–0.98)	0.04
	Abdominal pelvic pain	
Trimethoprim-sulfamethoxazole	6.75 (1.11–41.03)	0.03
	Pollakiuria	
Ciprofloxacin	0.21 (0.06–0.73)	0.01
	Treatment failure	
Amoxicillin-clavulanic acid	1.83 (1.03–3.26)	0.03
Cefalotin	2.08 (1.64–3.74)	0.01
Gentamicin	1.95 (1.05–3.61)	0.03
Ampicillin	0.048 (0.004–0.61)	0.02
	Recurrent UTIs	
Trimethoprim-sulfamethoxazole	18.75 (1.26–278.67)	0.03

In the paediatric population, abdominal-pelvic pain was associated with the bacterial phenotypes DTR-*E. coli* (*p* = 0.01), CRE-*E. coli* (*p* = 0.02) and XDR-*E. coli* (*p* = 0.03). While UDR-*K. pneumoniae* (*p* = 0.01) was significantly more frequent in urinary burning and neonates and infants (*p* = 0.02) (Fig. 1A, Table 4). We also found that DTR-*E. coli* (*p* = 0.04) was more common in urban areas (Fig. 1A, Table 4), while MDR-*E. coli* (*p* < 0.001), UDR-*E. coli* (*p* = 0.02), and ESC-*E. coli* (*p* < 0.001), were more frequently observed in male children (Fig. 1A, Table 4).

### Distribution of resistance phenotypes in *S. aureus* and *Enterococcus* spp

Among *S. aureus* and *Enterococcus* spp. isolates, MDR (86%, 37/43) and UDR (81%, 35/43) phenotypes were the most frequent. Among MDR strains, vancomycin-sensitive *Enterococcus* (VSE) (51%, 19/37) were significantly more frequent than the other resistance phenotypes (*p* < 0.0001). Similarly, the UDR phenotype was significantly associated with vancomycin-sensitive *Enterococcus* (VSE) (*p* = 0.01) (Table 6).

**Table 6:** Prevalence of major *ESKAPE*-Gram*-*positive uropathogens in resistance categories

	Resistance categories		
	MDR (n = 37)	XDR (n = 2)	UDR (n = 35)
MRSA	10 (27%)	1 (50%)	9 (26%)
MSSA	3 (8%)	0 (0%)	8 (23%)
VRE	5 (14%)	1 (50%)	3 (8%)
VSE	19 (51%)	0 (0%)	15 (43%)
Chi-squared	22.0	2.7	11.1
df	3	3	3
*P*-value	< 0.0001	NS	0.01

Statistical analyses were performed using a Chi-squared test in R software, version 4.0.2

*NS* not significant

FCA and Multivariate logistic regression analyses show that MDR- *Enterococcus* (*p* < 0.01) was associated with treatment failure (Fig. 1B, Table 4). In addition, MDR-*Enterococcus* (*p* = 0.04) and UDR-*Enterococcus* (*p* = 0.02) were more observed in male children (Fig. 1B, Table 4).

### Distribution of resistance to commonly used antibiotics in UTIs in children

The distribution of antibiotic resistance in *E. coli* and *K. pneumoniae* showed that bacterial resistance to Amoxicillin-clavulanic acid (*p* = 0.03), Cefalotin (*p* = 0.01), Ampicillin (*p* = 0.02) and Gentamicin (*p* = 0.03) were associated with treatment failure (Fig. 1A, Table 5) while the resistance to Ampicillin (*p* < 0.01), Cefotaxime (*p* = 0.04), Ciprofloxacin (*p* < 0.001) and Amikacin (*p* = 0.04) were more frequently observed among male children. Moreover, resistance to Trimethoprim-sulfamethoxazole (*p* = 0.03) was associated with abdomino-pelvic pain and recurrent UTIs (Fig. 1A, Table 5).

Bacterial resistance to the quinolone family such as Ciprofloxacin was associated with Pollakiuria (*p* = 0.01) and urinary burning (*p* = 0.04) (Fig. 1A, Table 5).

Among the major *ESKAPE*-Gram-positive, bacterial resistance to Ampicillin (*p* = 0.02) was associated with treatment failure (Fig. 1B, Table 5), while Benzylpenicillin-resistant bacteria (*p* = 0.03) were more common in male children (Fig. 1B, Table 5).

**Fig. 2:**
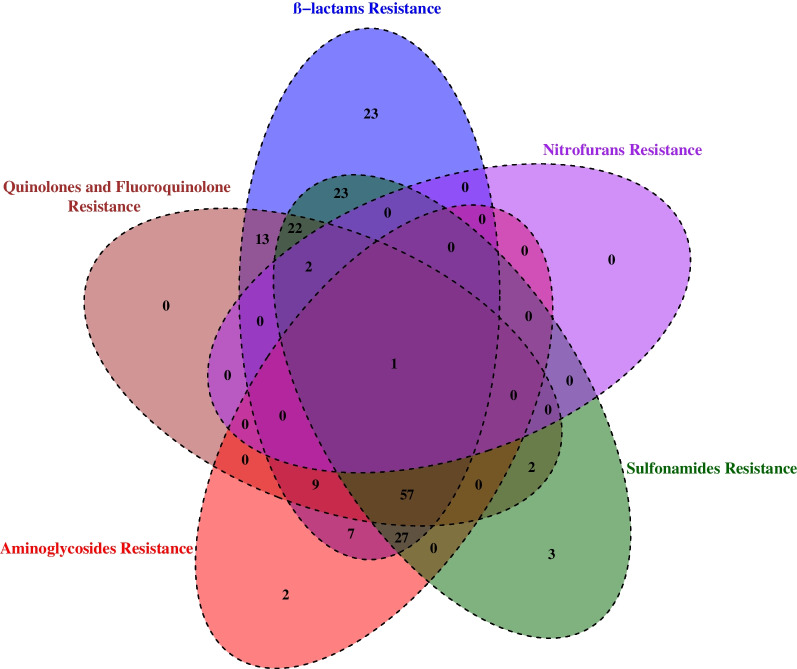
Venn diagram showing the distribution and co-occurrence of antibiotic resistance of the major *ESKAPE*-Gram-negative within the different antibiotic families. Each ellipse illustrates an antibiotic family for which resistance was assessed

In this study, Venn diagram was use to show the distribution and relationship between resistance of different families of antibiotics tested with *E. coli* and *K. pneumoniae* isolates (Fig. 2).

Overall, the highest rate of resistance was observed within the β-lactams family with 88% (184/209) followed by sulfonamides (65%, 137/209) and quinolones (50%, 106 /209) against 1% (3/209) observed for the family of nitrofurans (Fig. 2).

Venn diagram also reported that 11% (23/209), 0.95% (2/209) and 1.43% (3/209) of *E. coli* and *K. pneumoniae* isolates showed monoresistance to β-lactams, aminoglycosides and sulfonamides (Fig. 2).

The co-occurrence of resistance to two antibiotic families was predominantly observed with β-lactam/sulfonamide combination (63%, 132/209) (Fig. 2).

The co-occurrence of resistance to three antibiotic families was highest with the combination β-lactam-sulfonamide-aminoglycoside (12.9%, 27/209) while β-lactam-aminoglycoside-sulfonamide-quinolone combination showed the highest co-occurrence with four antibiotic families (27%, 57/209). Only one isolate showed resistance to all antibiotic families tested (Fig. 2).

### Multiple antibiotic resistance (MAR) index of *E. coli* and *Klebsiella* spp

The MAR index results showed that more than half of *E. coli* and *K. pneumoniae* isolates had resistance to at least 5 antibiotics (MAR index ≥ 0.26) while only one isolate showed resistance to 17 antibiotics (MAR index = 0.89) (Table 7).

**Table 7:** Multiple antibiotic resistance (MAR) index of *E. coli* and *K. pneumoniae*

MAR index	Isolate frequency (%)
0.00	24.1
0.05	4.0
0.10	2.0
0.15	5.0
0.21	5.0
0.26	11.0
0.31	6.0
0.36	4.0
0.42	10.0
0.47	7.0
0.52	6.0
0.57	4.0
0.63	2.0
0.68	5.0
0.73	2.0
0.78	2.0
0.89	0.9

MAR index was determined for each isolate using the formula MAR = n/N, where n represents the number of antibiotics to which the test isolate showed resistance and N represents the total number of antibiotics to which the test isolate has been evaluated. The MAR index ranged from 0.0 to 1.0

## Discussion

Several studies in resource-limited countries suggested that major pathogens found in urinary tract infections are often resistant to standard antibiotics [22], especially those isolated from UTIs in children [10]. The aim of this study was to determine the epidemiology and antibiotic susceptibility of major *ESKAPE* uropathogens in community-acquired paediatric UTIs in South-East Gabon.

The prevalence of urinary tract infections was 59%. They were significantly more frequent in neonates and infants (Table 1), corroborating the findings of Hay et al., who found a 46.6% rate of UTIs in children under 5 years [16].

*E. coli and K. pneumoniae* were the main uropathogenic *ESKAPE*s isolated in this study, with 35% and 34%, respectively, followed by *Enterococcus* spp. and *S. aureus* (Table 2). These results corroborate those of previous studies conducted in Africa [23–25]*.* Many virulence and fitness factors confer advantages to uropathogenic *E. coli* (UPEC) and *K. pneumoniae* (UPKP) in the host urinary tract. UPEC usually has a superficial viruloma and a secretome that contribute to its virulence and survival [26].

We describe the DTR and UDR resistance phenotypes as well as MAR index in Gabon (Tables 3, 7). These resistance phenotypes are now preferred to MDR, XDR and PDR phenotypes.

Indeed, MDR, XDR and PDR resistance phenotypes make no distinction between the strengths and weaknesses of each antibiotic: antibiotics with higher efficacy and lower toxicity are considered the same as those with lower efficacy and higher toxicity [27, 28]. Characterization of DTR, UDR, ESC, and CRE phenotypes facilitates resistance monitoring for clinicians (Tables 3, 6), as well as the improvement of empirical and targeted treatment regimens [27].

Among the major uropathogens, MDR resistance phenotype was the most common (Table 3). Similar rates of MDR uropathogenic strains have been previously described in Gabon [15]. However, a lower rate of MDR isolates was reported by Dikoumba et al. [29]. The differences observed could be explained by the origin of the samples used in each study.

MDR-*E. coli,* ESC-*E. coli,* MDR- *Enterococcus* were more frequently observed among male children. Male gender has already been described as a likely risk factor in the occurrence of MDR UTIs [20]. In the previously cited study, the authors showed that female gender was a protective factor in the occurrence of antibiotic-resistant UTIs, which support the results of the present study. The observation of multidrug-resistant phenotypes in male children could be a risk factor both for the failure of empirical treatment and for the aggravation of clinical forms. Indeed, male urinary tract infections present a high risk of complications because of the frequency of anatomical or functional abnormalities associated with them. [30].

DTR-*E. coli,* CRE-*E. coli*, XDR-*E. coli* were associated abdomino-pelvic pain and UDR-*K. pneumoniae* with urinary burning. Although the relationship between virulence and resistance appears to be antagonistic [31], authors have shown that one of the *E. coli* clones that globally disseminates extended-spectrum β-lactamases and NDM-1 carbapenemases, with few classical virulence factors, was virulent in a mouse model of sepsis [32, 33]. Thus, strains with virulence and resistance capabilities emerge as antibiotic selection pressure increases [34]. Other authors have provided epidemiological evidence that resistance and virulence phenotypes are linked in *E. coli* isolates of community origin [35]. Resistance phenotypes observed in this study strongly impact optimal care of community-based UTIs often treated by empirical antibiotherapy [36].

The presence of MDR, XDR, CRE, ESC, DTR, MRSA and VRE phenotypes may force clinicians to use antibiotics with less beneficial or limited pharmacological properties such as aminoglycosides (nephrotoxic, ototoxic and neurotoxic) and colistin (nephrotoxic and neurotoxic) [37].

In general, DTR-*E. coli* was more common in children from urban area. UTIs are quite common in community settings. However, many patients refuse to seek medical attention because of the social stigma associated with UTIs. In urban areas, the lack of paediatricians also leads to long queues during medical consultations, discouraging many patients who opt for self-medication, largely responsible for the selection of resistant strains. Also, the lack of education and the uncontrolled sale of counterfeit or substandard antibiotics are two additional factors favouring the emergence and dissemination of bacterial resistance in urban UTIs.

Multivariate logistic regression analysis showed associations between several antibiotics with patients’ socioclinical parameters. Indeed, Male gender was associated with resistance to β-lactams (Benzylpenicillin, Ampicillin and Cefotaxime), fluoroquinolones (Ciprofloxacin) and aminoglycosides (Amikacin) as previously reported by Shaikh et al. [38].

Children age was associated with β-lactams-resistance. As the resistance to Benzylpenicillin, Cefalotin and Cefotaxime was more frequent in neonates and infants, while those to Cefalotin and Cefotaxime were more common in early and late chilhood.

Another clinical symptoms was associated with antimicrobial resistance. Abdominopelvic pain-like symptoms were associated with resistance to sulfonamides (Trimethoprim-sulfamethoxazole) while urinary burning and pollakiuria were associated with resistance to fluoroquinolones (Ciprofloxacin). Our results are in accordance of a previous report which found an association between clinical signs and antibiotic resistance in *E. coli* isolated from pediatric UTIs [39].

Furthermore, recurrent UTIs were associated with resistance to sulfonamides (Trimethoprim-sulfamethoxazole) as also reported by Nelson et al. [40].

Finally, treatment failure in children was associated with resistance to β-lactams (Ampicillin, Amoxicillin-clavulanic acid, Cefalotin) and aminoglycosides (Gentamicin).

In this study, the frequency of DTR, ESC, CRE, MDR, MRSA and ERV phenotypes was not negligible in paediatric UTIs. However, the high presence of the UDR phenotype (more than 50%) in major uropathogens suggests that these infections remain treatable with standard antibiotics.

## Conclusion

This study describes for the first time the resistance phenotypes DTR, UDR and MAR index in Gabon. The prevalence of UTIs was high with a strong involvement of the uropathogens *E. coli*, *K. pneumoniae*, *Enterococcus* spp. and *S. aureus*. Neonates and infants were predominantly affected by UTIs. The study also showed that the majority of paediatric UTIs remain susceptible to standard antibiotic therapy. However, the presence of MDR, XDR, DTR, ESC, CRE, MRSA and VRE phenotypes in the paediatric population should alert health authorities to the need to set up a national surveillance system for antimicrobial resistance in Gabon.

## Data Availability

Data and materials supporting the conclusions of this study will be made available on request to the corresponding author.

## Abbreviations

ESKAPE: *Enterococcus faecalis*, *Staphylococcus aureus*, *Klebsiella pneumoniae, Acinetobacter baumannii*, *Pseudomonas*, *Enterobacteriaceae*
IVUs: Infection des voies urinaires
MDR: Multidrug resistance
XDR: Extensive drug resistance
PDR: Pandrug-resistance
DTR: Difficult to Treat Resistance
UDR: Usual drug resistance
ESC: Extended-spectrum cephalosporin-resistant
CRE: Carbapenem-resistant *Enterobacterales*
MAR: Multiple antibiotic resistance
SARM: Methicillin-resistant* Staphylococcus aureus*
SASM: Methicillin-sensitive* Staphylococcus aureus*
VRE: Vancomycin-resistant *Enterococcus*
VSE: Vancomycin-sensitive *Enterococcus*
UPEC: Uropathogenic-*E. coli*
UPKP: Uropathogenic*-K. pneumoniae*
CFU: Colony-forming units
